# Machine learning-driven risk stratification for distant metastasis in gastric cancer: A comparative study of clinical features and composite indices integrated models

**DOI:** 10.1371/journal.pone.0335258

**Published:** 2025-10-30

**Authors:** Shaoxue Yang, Han Lei

**Affiliations:** 1 Department of Laboratory Medicine, Zhejiang Cancer Hospital, Hangzhou, Zhejiang, People’s Republic of China; 2 Department of Laboratory Medicine, The First Affiliated Hospital of Zhejiang Chinese Medical University (Zhejiang Provincial Hospital of Chinese Medicine), Hangzhou, Zhejiang, People’s Republic of China; Hokkaido University: Hokkaido Daigaku, JAPAN

## Abstract

**Objective:**

Distant metastasis (DM) of gastric cancer (GC) represents a significant health challenge due to its high mortality rates, necessitating advancements in early detection and management strategies. The objective of this study was to create a machine learning (ML) model that is interpretable for preoperative prediction of DM in GC.

**Methods:**

We retrospectively analyzed 1,009 GC patients, of which 769 were from Zhejiang Cancer Hospital as development cohort and 240 from Zhejiang Provincial Hospital of Chinese Medicine as external test cohort. Nine clinical features, and four composite indices derived from ten laboratory indicators were selected as candidate features. The dataset was balanced using the borderline Synthetic Minority Over-sampling Technique (SMOTE) and the Edited Nearest Neighbors (ENN) under-sampling method. Univariate and multivariate analyses were used to identified key metastasis-related features. Based on the identified features, we developed predictive models incorporating five ML algorithms, with performance evaluated via receive operating characteristic (ROC) curves, recall, precision-recall (PR) curves. Ultimately, Shapley additive explanations (SHAP) analysis were applied to rank the feature importance and explain the final model.

**Results:**

Univariate and multivariate analyses identified five metastasis-related features: cT stage, cN stage, differentiation grade, PLR and TMI. Logistic Regression emerged as the optimal predictive model with the highest area under the curve (AUC) of 0.942 (95% CI: 0.922–0.962), Recall of 0.895 (95% CI: 0.843–0.947), and AUPRC of 0.889 (95% CI: 0.867–0.911) among five models. Additionally, the internal and external test cohorts recorded AUC values of 0.935 (95% CI: 0.897–0.972) and 0.879 (95% CI: 0.833–0.926), respectively. The SHAP analysis revealed the features that played a significant role in the predictions made by the model.

**Conclusion:**

This ML model integrates clinical features and composite indices to predict GC metastasis risk, supported by an online tool to guide preoperative decision-making.

## Introduction

Gastric cancer (GC) is a significant global health concern, ranking fifth in incidence and fourth in mortality worldwide. In China, 24.05% of individuals diagnosed with GC have distant metastases (DM). The peritoneum, liver, and bones are the three most frequent sites of metastatic spread [1–2]. The therapeutic approach for metastatic disease fundamentally differs from that of non-metastatic disease, transitioning from endoscopic resection or curative-intent gastrectomy to palliative systemic therapy, such as chemotherapy, molecularly targeted therapies, and immunotherapeutic regimens [3–4]. This distinction highlights the critical importance of accurately identifying the metastatic status to guide appropriate clinical management.

Detecting DM in GC remains challenging due to the limitations of existing technologies. For example, CT/PET-CT has low sensitivity for identifying sub-5 mm peritoneal micrometastases and often fails to differentiate metastatic lesions from inflammatory changes, particularly in hypometabolic tumors [5–6]. Liquid biopsies, which include circulating tumor DNA and circulating tumor cells, show promise but suffer from low sensitivity in the early detection of metastasis and lack standardized protocols. Tumor heterogeneity further compromises their reliability [7–9]. The substantial costs associated with PET-CT and liquid biopsies impose significant financial burdens on patients, limiting their feasibility for routine monitoring applications.

Machine learning (ML) is revolutionizing disease prediction by integrating diverse data sources to enable earlier and more precise risk assessment. These technologies excel in chronic disease management, oncology, infectious disease surveillance, and rare disease diagnosis [10–15]. The majority of current ML models for predicting GC with DM are based on the Surveillance, Epidemiology, and End Results (SEER) database. These models predominantly leverage demographic characteristics and clinicopathological profiles, rarely integrating multimodal predictors with laboratory-derived composite indices into their predictive architectures [16–19]. Compared to single laboratory indicators, accumulating evidence demonstrates that composite indices play significant roles in tumor early detection and prognostication. Neutrophil-to-lymphocyte ratio (NLR), platelet-to-lymphocyte ratio (PLR), platelet-to-monocyte ratio (PMR), and systemic immune-inflammation index (SIII) are common indicators of systemic inflammation, which have been identified as potential predictors for prognosis and treatment efficacy evaluation in various types of cancer [20–23]. Tumor Marker Index (TMI) and Prognostic Nutritional Index (PNI) are recognized as important prognostic factors in multiple malignancies [24–27]. However, limited studies have applied these composite indices to the investigation of DM in GC.

In this research, our objective was to create and assess an optimal explainable ML model for preoperative forecasting risk of DM in GC using clinical features and laboratory-derived composite indices, thereby assisting clinicians in reducing unnecessary surgical trauma caused by inappropriate radical surgery for patients at high DM risk.

## Methods

### Patients involvement

**Fig 1:**
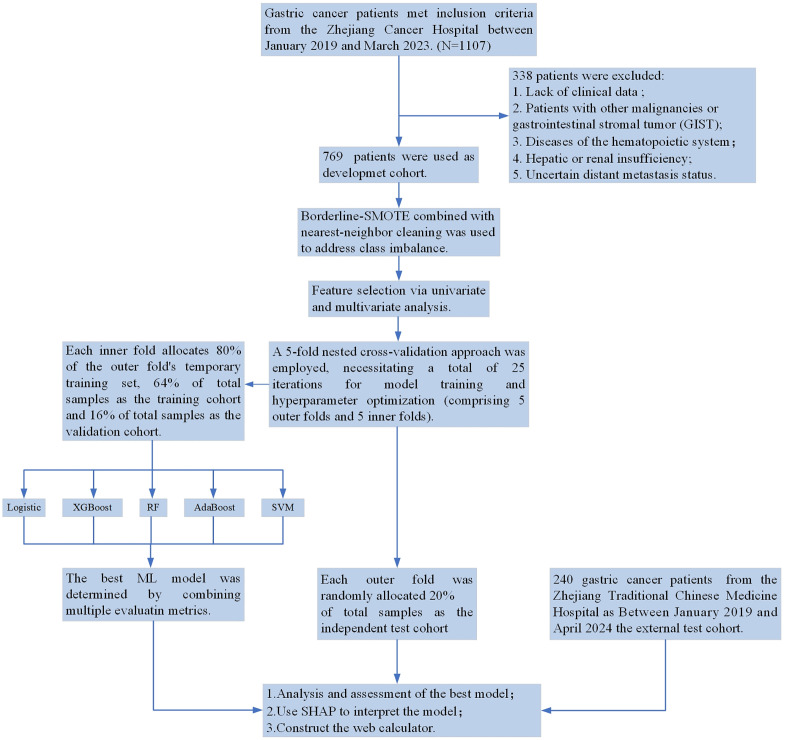
The workflow diagram for study design and patient screening. Abbreviations: SMOTE: Synthetic Minority Oversampling Technique; XGBoost: eXtreme Gradient Boosting; RF: Random Forest; AdaBoost, Adaptive Boosting; SVM: Support Vector Machine; SHAP: Shapley additive explanations.

In this retrospective study, all data were extracted from the electronic medical record system (EMRS) following ethical approval from both the Ethics Committee of Zhejiang Cancer Hospital (No. IRB-2022–140), with initial access commencing on September 30, 2022; and the Ethics Committee of the First Affiliated Hospital of Zhejiang Chinese Medical University (No. 2023-KLS-137–01), with initial access commencing on October 20, 2023. Minors were not included in this study. This retrospective study utilized fully anonymized medical record data. The ethics committee waived the requirement for informed consent, and all procedures adhered to the Declaration of Helsinki. A total of 1,107 GC patients treated at Zhejiang Cancer Hospital were enrolled between January 2019 and March 2023. After excluding certain patients, 769 patients were included in the final analysis as the development cohort. The inclusion criteria were as follows: (1) pathological diagnosis of gastric adenocarcinoma; (2) preoperative patients who had not received neoadjuvant therapy or radiation; (3) non-residual gastric cancer. The exclusion criteria were: (1) lack of clinical data; (2) patients with other malignancies or gastrointestinal stromal tumor (GIST); (3) diseases of the hematopoietic system; (4) hepatic or renal insufficiency; (5) uncertain distant metastasis status. The external test cohort comprised 240 GC patients from the Zhejiang Traditional Chinese Medicine Hospital, all of whom met the same inclusion and exclusion criteria. The patients were treated between January 2019 and April 2024. The study flow of this paper is shown in Fig 1.

### Data collection and processing

The collection of clinical features encompassed gender, age, tumor location, tumor size, Lauren classification, differentiation grade, clinical T stage, clinical N stage, and Her2 status. Laboratory indicators comprised lymphocyte count, monocyte count, platelet count, albumin level, CA242, CA50, CA724, CEA, CA125, and CA199 levels. All data were obtained from the EMRS and were collected prior to treatment initiation. The clinical tumor stage and DM were classified based on the 8th edition of the AJCC Cancer Staging System. The PLR, LMR, PNI and TMI were calculated as follows: PLR = Platelet count (10^9^/L) ÷ Lymphocyte count (10^9^/L); LMR = Lymphocyte count (10^9^/L) ÷ Monocyte count (10^9^/L); PNI = Albumin (g/L) + 5 × Lymphocyte count (10^9^/L); the calculation method for TMI was proposed by Miyata T et al [28], and TMI was defined as the number of positive TMs for individual, the value exceeding the upper limit of the reference range is considered positive, and the upper limit of CA50, CEA, CA19−9, CA12−5, CA72−4, CA24−2 were 25 U/mL, 5 ng/mL, 34 U/mL, 35 U/mL, 6.9 U/mL and 20 U/mL, respectively.

### Sample balancing strategies

Considering the class imbalance in the gastric cancer (GC) metastasis dataset (with metastatic cases constituting only 18.9% and non-metastatic cases making up 81.1%), the borderline Synthetic Minority Over-sampling Technique (SMOTE) and the Edited Nearest Neighbors (ENN) under-sampling method was employed to address the imbalance. First, the borderline SMOTE algorithm was used for data augmentation. This method generated new minority-class (metastatic) samples by leveraging feature similarities among existing positive cases. Subsequently, the ENN cleaning approach was implemented to remove majority-class (non-metastatic) samples that overlapped with minority-class samples. These overlapping cases are prevalent noise sources in imbalanced datasets. This strategy not only enhances model training accuracy but also better reflects class-specific sample distributions while avoiding the noise associated with synthetic data.

### Feature selection and validation strategy

To select optimal features for predicting gastric cancer metastasis, we first applied univariate analysis to filter out variables with weak associations (P > 0.05). The remaining candidates were then included in multivariate stepwise regression to eliminate collinearity and retain only those with independent predictive value (P < 0.05). To evaluate the model performance, we employed five-fold nested cross-validation. Five-fold nested cross-validation is a robust validation method that uses double-layer data partitioning. The outer loop evaluates model performance by dividing the development data into four training folds and one test fold, while the inner loop optimizes hyperparameters through five splits, utilizing four subsets for training and one for validation. This approach prevents data leakage, ensures unbiased assessment, and improves generalizability, although it requires 25 training iterations. The optimal hyperparameters for the model were determined using a grid search approach, which systematically assesses each and every combination of hyperparameters. The internal and external test cohorts were employed to validate the final optimal model derived through rigorous filtering.

### ML algorithms

The optimal ML model was selected from a pool of five algorithms: eXtreme Gradient Boosting (XGBoost), Logistic Regression, Random Forest (RF), Adaptive Boosting (AdaBoost), and Support Vector Machine (SVM). Subsequently, ROC curves, precision-recall (PR) curves, calibration curves, and decision curves analysis (DCA) were used to evaluate the models’ performance. A battery of metrics including AUC, accuracy, recall, specificity, PRAUC, Brier and F1 score were employed to assess the models. Following a thorough comparative analysis of the various ML models, the one exhibiting the best AUC, recall and PRAUC in validation cohort was selected as the final predictive model. Ultimately, Shapley additive explanations (SHAP) analysis were utilized to interpret and evaluate the final model.

### Statistical analysis

Statistical evaluations were conducted utilizing Python version 3.11.4 and R version 4.2.3. Categorical variables are represented as percentages, while continuous variables are denoted as mean ± standard deviation or median accompanied by the interquartile range (IQR). For the analysis of categorical data, the Chi-square test was employed, whereas the assessment of continuous variables was carried out using either Student’s t-test or the Mann–Whitney U test were used to analyze continuous data. ROC curves were conducted to evaluate the discrimination performance of the models, including the AUCs along with their 95% confidence interval (CI) being reported. The Brier score, which varies between 0 and 1, was employed to evaluate the discrepancy between the predicted risk and the actual risk, where a score approaching 0 signifies superior calibration and serves as a measure of model calibration. Additionally, DCA was performed to illustrate the net benefit derived from utilizing the model at varying thresholds, thereby assessing the model’s clinical relevance. A two-tailed P-value of less than 0.05 was deemed statistically significant.

## Results

### Baseline clinical characteristics

The essential demographic and clinical characteristics of GC patients from the two centers are detailed in Table 1. A total of 769 patients from Zhejiang Cancer Hospital were classified into the development cohort, while 240 patients from Zhejiang Provincial Hospital of Traditional Chinese Medicine comprised the external test cohort. Statistical analysis revealed no significant differences in gender, age, metastasis category, or metastasis sites between the cohorts (all P > 0.05), confirming comparable baseline characteristics. Table 2 presents the baseline clinical characteristics of the development cohort, which included 145 patients with DM and 624 non-metastatic patients. The groups showed no significant differences in gender, age, HER2 status, or monocyte count (all P > 0.05), whereas statistically significant variations were observed in tumor location, tumor size, Lauren classification, clinical T/N stages, lymphocyte count, platelet count, albumin, CA242, CA50, CA724, CEA, CA125, CA199, PLR, LMR, PNI, and TMI (all P < 0.05).

**Table 1:** Baseline characteristics of gastric gancer cohorts from two centers.

Characteristics	Development cohort (N = 769)	External test cohort (N = 240)	P-value
Gender, n (%)			
Male	510(66.32)	167(69.58)	0.348
Female	259(33.68)	73(30.42)	
Age, n(%)			
≤ 64 years	386(50.20)	112(46.67)	0.340
> 64 years	383(49.80)	128(53.33)	
Metastasis Category, n (%)			
M0	624(81.14)	194(80.83)	0.914
M1	145(18.86)	46(19.17)	
Metastasis site, n (%)			
Peritoneum	39(26.90)	12(26.09)	0.914
Liver	35(24.14)	10(21.74)	0.738
Lung	12(8.28)	6(13.04)	0.335
Ovary	11(7.58)	3(6.52)	0.809
Bone	11(7.58)	4(8.70)	0.807
Distant lymph node	7(4.83)	2(4.35)	0.894
Other	6(4.14)	1(2.17)	0.537
Multiple	24(16.55)	8(17.39)	0.894

M0: patients without distant metastasis; M1: patients with distant metastasis; Other: less common metastatic sites, such as bladder, adrenal, pancreas; Multiple: two or more distant metastasis sites.

**Table 2:** Comparison of characteristics between Mo and M1 gastric cancer patients from development cohort.

Characteristics	ALL (N = 769)	M0 (N = 624)	M1 (N = 145)	P-value
Gender, n (%)				
Male	510 (66.32)	418 (66.99)	92 (63.45)	0.417
Female	259 (33.68)	206 (33.01)	53 (36.55)	
Age, n (%)				
≤ 64 years	386 (50.20)	315 (50.48)	71 (48.97)	0.742
> 64 years	383 (49.81)	309 (49.52)	74 (51.03)	
Location, n (%)				
Upper third	117 (15.22)	95 (15.22)	22 (15.17)	**0.011**
Middle third	253 (32.90)	219 (35.10)	34 (23.45)	
Lower third	363 (47.20)	286 (45.83)	77 (53.10)	
Entire	36 (4.68)	24 (3.85)	12 (8.28)	
Size, n (%)				
< 2 cm	171 (22.24)	165 (26.44)	6 (4.14)	**<0.001**
2-5 cm	399 (51.89)	334 (53.53)	65 (44.83)	
> 5 cm	199 (25.88)	125 (20.03)	74 (51.03)	
Lauren classification, n (%)				
Diffuse	295 (38.36)	224 (35.90)	71 (48.96)	**0.008**
Intestinal	292 (37.97)	251 (40.22)	41 (28.28)	
Mixed	182 (23.67)	149 (23.88)	33 (22.76)	
Differentiation grade, n (%)				
G3, G4	350 (45.51)	261 (41.83)	89 (61.38)	**<0.001**
G2-G3, G2	300 (39.01)	257 (41.19)	43 (29.65)	
G1-G2, G1	119 (15.48)	106 (16.98)	13 (8.97)	
Clinical T stage, n (%)				
T1	217 (28.22)	216 (34.62)	1 (0.69)	**< 0.001**
T2	93 (12.09)	88 (14.10)	5 (3.45)	
T3	166 (21.59)	132 (21.15)	34 (23.45)	
T4	293 (38.10)	188 (30.13)	105 (72.41)	
Clinical N stage, n (%)				
N0	281 (36.54)	277 (44.39)	4 (2.76)	**< 0.001**
N1	139 (18.08)	120 (19.23)	19 (13.10)	
N2	160 (20.81)	120 (19.23)	40 (27.59)	
N3	189 (24.58)	107 (17.15)	82 (56.55)	
Her2, n (%)				
0	303 (39.40)	241 (38.62)	62 (42.76)	0.778
1+	231 (30.04)	188 (30.13)	43 (29.65)	
2+	197 (25.62)	164 (26.28)	33 (22.76)	
3+	38 (4.94)	31 (4.97)	7 (4.83)	
Lymphocyt〔median(IQR), 109/L〕	1.5 (1.2, 1.8)	1.5 (1.2, 1.8)	1.4 (1.1, 1.7)	**0.002**
Monocyte〔median (IQR), 109/L〕	0.39 (0.39, 0.52)	0.39 (0.39, 0.52)	0.52 (0.39, 0.65)	0.165
Platelet〔median (IQR), 109/L〕	237 (195, 290)	232 (195, 282)	259 (204, 320)	**<0.001**
Albumin〔median (IQR), g/L〕	38.1 (34.9, 41.4)	38.5 (35.3, 41.6)	37.0 (33.7, 40.0)	**<0.001**
CA24−2〔median (IQR), U/mL〕	4.06 (2.35, 7.79)	3.73 (2.20, 6.31)	8.73 (3.46, 105.00)	**<0.001**
CA50〔median (IQR), U/mL〕	6.11 (4.03, 11.30)	5.48 (3.78, 9.12)	16.20 (6.19, 112.00)	**<0.001**
CA72−4〔median (IQR), U/mL〕	2.09 (1.50, 5.33)	1.81 (1.40, 4.12)	5.53 (2.09, 16.93)	**<0.001**
CEA〔median(IQR), ng/mL〕	1.62 (0.83, 3.12)	1.49 (0.78, 2.70)	2.65 (1.16, 5.69)	**<0.001**
CA12−5〔median (IQR), U/mL〕	13.07 (9.38, 19.68)	12.30 (9.10, 17.30)	20.13 (12.06, 34.50)	**<0.001**
CA19−9〔median (IQR), U/mL〕	13.50 (7.27, 24.67)	12.05 (6.66, 19.76)	37.93 (12.64, 350.91)	**<0.001**
PLR〔median (IQR)〕	155.00 (119.00, 216.00)	148.67 (114.62, 203.53)	194.00 (149.10, 255.00)	**<0.001**
LMR〔median (IQR)〕	3.46 (2.56, 4.62)	3.54 (2.69, 4.62)	3.08 (2.18, 4.10)	**<0.001**
PNI〔median (IQR)〕	45.94 (41.84, 49.59)	46.40 (42.41, 50.06)	43.77 (39.91, 47.66)	**<0.001**
TMI〔median (IQR)〕	0 (0, 1)	0 (0, 0)	2 (1, 4)	**<0.001**

M0: patients without distant metastasis; M1: patients with distant metastasis; G1:Well differentiated; G1-G2: Well-Moderately differentiated; G2: Moderately differentiated; G2-G3: Moderately-poorly differentiated; G3: Poorly differentiated; G4: Undifferentiated; PLR: Platelet-to-lymphocyte ratio; LMR: Lymphocyte-to-monocyte ratio; PNI: Prognostic nutritional index; TMI: Tumor marker index.

### Sample balancing via borderline SMOTE-ENN method

After sample balancing, metastasis samples increased from 145 to 250 (preserving clinical feature consistency), no metastasis samples samples decreased from 624 to 500 (clean invalid noise). The resultant 750-sample dataset (250 positive, 500 negative) maintained a 1:2 ratio. No significant feature distribution differences from the original dataset (all P > 0.05) confirmed no data distortion, as shown in Table 3.

**Table 3:** Comparison of clinical characteristics between original cohort and borderline SMOTE-ENN balanced cohort in M0 and M1 gastric cancer.

Characteristics	M0		P-value	M1		P-value
	Original cohort (N = 624)	balanced cohort (N = 500)		Original cohort (N = 145)	balanced cohort (N = 250)	
Gender, n (%)			0.620			0.747
Male	418 (66.99)	327 (65.40)		92 (63.45)	164 (65.60)	
Female	206 (33.01)	173 (34.60)		53 (36.55)	86 (34.40)	
Age, n (%)			0.655			0.213
≤ 64 years	315 (50.48)	260 (52.00)		71 (48.97)	140 (56.00)	
> 64 years	309 (49.52)	240 (48.00)		74 (51.03)	110 (44.00)	
Location, n (%)			0.913			0.087
Upper third	95 (15.22)	69 (13.80)		22 (15.17)	49 (19.60)	
Middle third	219 (35.10)	179 (35.80)		34 (23.45)	79 (31.60)	
Lower third	286 (45.83)	234 (46.80)		77 (53.10)	110 (44.00)	
Entire	24 (3.85)	18 (3.60)		12 (8.28)	12 (4.80)	
Size, n (%)			0.344			0.805
< 2 cm	165 (26.44)	146 (29.20)		6 (4.14)	11 (4.40)	
2-5 cm	334 (53.53)	269 (53.80)		65 (44.83)	120 (48.00)	
> 5 cm	125 (20.03)	85 (17.00)		74 (51.03)	119 (47.60)	
Lauren classification, n (%)			0.934			0.932
Diffuse	224 (35.90)	184 (36.80)		71 (48.96)	127 (50.80)	
Intestinal	251 (40.22)	196 (39.20)		41 (28.28)	67 (26.80)	
Mixed	149 (23.88)	120 (24.00)		33 (22.76)	56 (22.40)	
Differentiation grade, n (%)			0.906			0.414
G3, G4	261 (41.83)	212 (42.40)		89 (61.38)	166 (66.40)	
G2-G3,G2	257 (41.19)	208 (41.60)		43 (29.65)	59 (23.60)	
G1-G2,G1	106 (16.98)	80 (16.00)		13 (8.97)	25 (10.00)	
Clinical T stage, n (%)			0.390			0.253
T1	216 (34.62)	194 (38.80)		1 (0.69)	1 (0.40)	
T2	88 (14.10)	71 (14.20)		5 (3.45)	10 (4.00)	
T3	132 (21.15)	105 (21.00)		34 (23.45)	80 (32.00)	
T4	188 (30.13)	130 (26.00)		105 (72.41)	159 (63.60)	
Clinical N stage, n (%)			0.889			0.389
N0	277 (44.39)	227 (45.40)		4 (2.76)	7 (2.80)	
N1	120 (19.23)	97 (19.40)		19 (13.10)	40 (16.00)	
N2	120 (19.23)	99 (19.80)		40 (27.59)	84 (33.60)	
N3	107 (17.15)	77 (15.40)		82 (56.55)	119 (47.60)	
Her2, n (%)			0.824			0.140
0	241 (38.62)	195 (39.00)		62 (42.76)	113 (45.20)	
1+	188 (30.13)	154 (30.80)		43 (29.65)	92 (36.80)	
2+	164 (26.28)	132 (26.40)		33 (22.76)	38 (15.20)	
3+	31 (4.97)	19 (3.80)		7 (4.83)	7 (2.80)	
Lymphocyte〔median (IQR), 109/L〕	1.5 (1.2, 1.8)	1.6 (1.3, 1.8)	0.175	1.4 (1.1, 1.7)	1.4 (1.1, 1.6)	0.835
Monocyte〔median (IQR), 109/L〕	0.39 (0.39, 0.52)	0.39 (0.39, 0.52)	0.490	0.52 (0.39, 0.65)	0.46 (0.39, 0.58)	0.866
Platelet〔median (IQR), 109/L〕	232 (195, 282)	233 (196, 275)	0.902	259 (204, 320)	265 (216, 323)	0.366
Albumin〔median (IQR), g/L〕	38.5 (35.3, 41.6)	39.0 (35.7, 41.9)	0.093	37.0 (33.7, 40.0)	36.5 (34.1, 39.5)	0.740
CA24−2〔median (IQR), U/mL〕	3.73 (2.20, 6.31)	3.52 (2.15, 5.59)	0.156	8.73 (3.46, 105.00)	6.95 (3.46, 35.44)	0.168
CA50〔median (IQR), U/mL〕	5.48 (3.78, 9.12)	5.32 (3.73, 8.25)	0.100	16.20 (6.19, 112.00)	12.30 (6.29, 54.88)	0.143
CA72−4〔median (IQR), U/mL〕	1.81 (1.40, 4.12)	1.72 (1.34, 3.85)	0.315	5.53 (2.09, 16.93)	6.51 (2.31, 20.10)	0.746
CEA〔median (IQR), ng/mL〕	1.49 (0.78, 2.70)	1.41 (0.71, 2.53)	0.240	2.65 (1.16, 5.69)	2.391 (1.30, 5.25)	0.870
CA12−5〔median (IQR), U/mL〕	12.30 (9.10, 17.30)	11.93 (8.97, 16.49)	0.213	20.13 (12.06, 34.50)	19.68 (12.19, 31.15)	0.875
CA19−9〔median (IQR), U/mL〕	12.05 (6.66, 19.76)	11.23 (6.57, 17.77)	0.117	37.93 (12.64, 350.91)	28.61 (11.76, 160.71)	0.108
PLR〔median (IQR)〕	148.67 (114.62, 203.53)	146.46 (115.31, 193.93)	0.348	194.00 (149.10, 255.00)	198.62 (153.42, 253.70)	0.403
LMR〔median (IQR)〕	3.54 (2.69, 4.62)	3.59 (2.82, 4.62)	0.111	3.08 (2.18, 4.10)	3.08 (2.31, 3.85)	0.766
PNI〔median (IQR)〕	46.40 (42.41, 50.06)	47.12 (43.08, 50.48)	0.090	43.77 (39.91, 47.66)	43.49 (40.30, 46.68)	0.728
TMI〔median (IQR)〕	0 (0, 0)	0 (0, 0)	0.220	2 (1, 4)	2 (1, 3)	0.170

M0: patients without distant metastasis; M1: patients with distant metastasis; G1:Well differentiated; G1-G2: Well-Moderately differentiated; G2: Moderately differentiated; G2-G3: Moderately-poorly differentiated; G3: Poorly differentiated; G4: Undifferentiated; PLR: Platelet-to-lymphocyte ratio; LMR: Lymphocyte-to-monocyte ratio; PNI: Prognostic nutritional index; TMI: Tumor marker index.

### Feature selection associated with distant metastasis in gastric cancer

Univariate and multivariate analyses were performed to screen feature variables, and five key variables were ultimately identified: cT stage, cN stage, differentiation grade, PLR, and TMI. Detailed information on these variables is presented in Table 4.

**Table 4:** Univariate and multivariate analysis of factors associated with distant metastasis in gastric cancer.

Characteristics	Descript	P-value (univariable)	95% CI (univariable)	P-value (multivariable)	95% CI (multivariable)
Gender	Male				
	Female	0.957	0.99 (0.72 - 1.36)		
Age	≤ 64 years				
	> 64 years	0.301	0.85 (0.63 - 1.15)		
Location	Upper third				
	Middle third	**0.039**	0.62 (0.40 - 0.98)	0.297	0.65 (0.29 - 1.45)
	Lower third	**0.06**	0.66 (0.43 - 1.02)	0.294	0.68 (0.33 - 1.40)
	Entire	0.879	0.94 (0.41 - 2.13)	0.041	0.26 (0.07 - 0.95)
Size	< 2 cm				
	2-5 cm	**<0.001**	5.92 (3.09 - 11.34)	0.659	1.28 (0.42 - 3.89)
	> 5 cm	**<0.001**	18.58 (9.48 - 36.42)	0.174	2.28 (0.70 - 7.44)
Lauren classification	Diffuse				
	Intestinal	**<0.001**	0.50 (0.35 - 0.71)	0.866	0.94 (0.44 - 2.01)
	Mixed	**0.049**	0.68 (0.46 - 1.00)	0.502	1.29 (0.61 - 2.75)
Differentiation grade	G3, G4				
	G2-G3, G2	**<0.001**	0.36 (0.25 - 0.52)	**0.009**	0.38 (0.18 - 0.78)
	G1-G2, G1	**<0.001**	0.40 (0.24 - 0.65)	0.408	1.51 (0.57 - 3.97)
Clinical T stage	T1 + T2				
	T3	**<0.001**	18.35 (9.40 - 35.85)	**0.005**	4.79 (1.60 - 14.33)
	T4	**<0.001**	29.47 (15.44 - 56.21)	**<0.001**	6.77 (2.26 - 20.30)
Clinical N stage	N0				
	N1	**<0.001**	13.37 (5.79 - 30.90)	**<0.001**	10.43 (2.64 - 41.23)
	N2	**<0.001**	27.52 (12.28 - 61.63)	**<0.001**	15.39 (3.98 - 59.49)
	N3	**<0.001**	50.12 (22.41 - 112.09)	**<0.001**	26.58 (6.74 - 104.82)
Her2	0				
	1+	0.863	1.03 (0.73 - 1.46)	0.626	1.15 (0.65 - 2.04)
	2+	0.001	0.50 (0.32 - 0.76)	0.08	0.48 (0.21 - 1.09)
	3+	0.322	0.64 (0.26 - 1.56)	0.042	0.20 (0.04 - 0.94)
PLR	Median (IQR)	**<0.001**	1.01 (1.01 - 1.01)	**<0.001**	1.01 (1.01 - 1.01)
LMR	Median (IQR)	**<0.001**	0.71 (0.63 - 0.80)	0.439	1.08 (0.89 - 1.32)
PNI	Median (IQR)	**<0.001**	0.90 (0.87 - 0.92)	0.352	1.03 (0.97 - 1.10)
TMI	Median (IQR)	**<0.001**	5.23 (4.03 - 6.79)	**<0.001**	5.02 (3.66 - 6.89)

M0: patients without distant metastasis; M1: patients with distant metastasis; G1:Well differentiated; G1-G2: Well-Moderately differentiated; G2: Moderately differentiated; G2-G3: Moderately-poorly differentiated; G3: Poorly differentiated; G4: Undifferentiated; PLR: Platelet-to-lymphocyte ratio; LMR: Lymphocyte-to-monocyte ratio; PNI: Prognostic nutritional index; TMI: Tumor marker index.

### Identification of the optimal ML model

**Fig 2:**
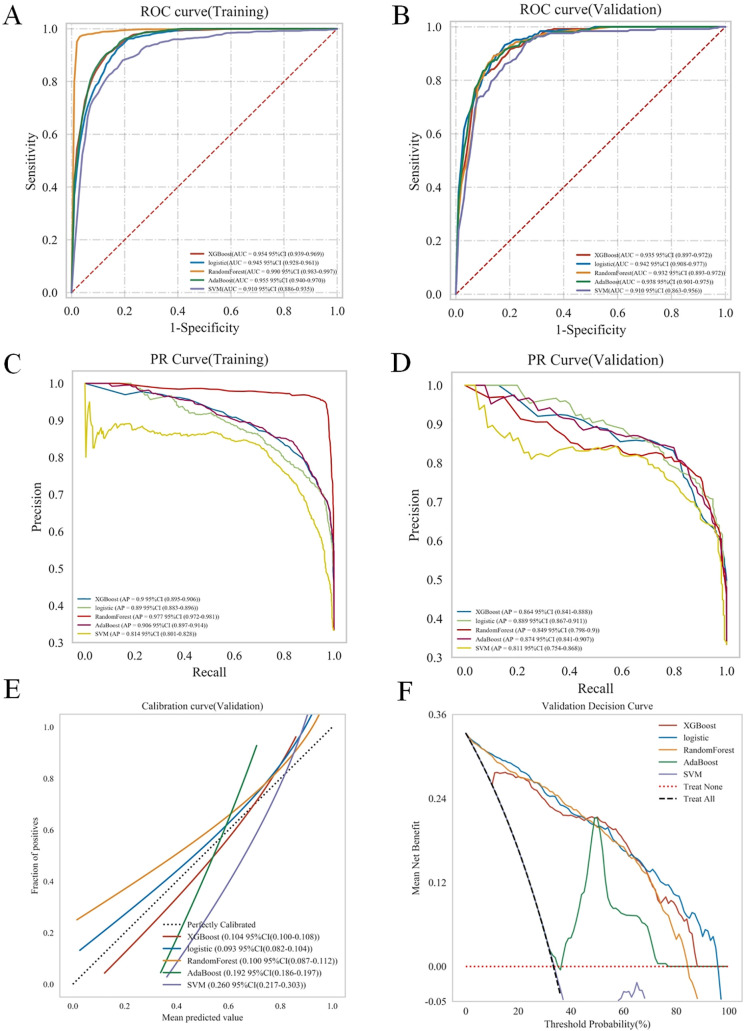
The performance of five-model in forecasting distant metastasis among gastric cancer patients, as evaluated in both the training and validation cohorts. The analysis of the ROC curves (A, B) and the PR curves (C, D) were conducted for each model within both the training and validation cohorts. Calibration curves (E) and DCA curves (F) for each model in the validation cohorts. Abbreviations: XGBoost: eXtreme Gradient Boosting; AdaBoost, Adaptive Boosting; SVM: Support Vector Machine.

**Fig 3:**
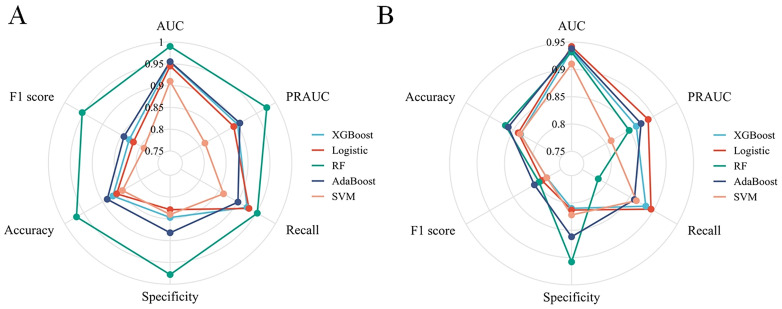
Five-model comparative analysis via radar plot: AUC, accuracy, sensitivity, specificity, F1-Score, AUPRC evaluation. (A) Comparative analysis in the training cohort. (B) Comparative analysis in the validation cohort. Abbreviations: AUC: area under the curve; AUPRC: area under the precision-recall curve; XGBoost: eXtreme Gradient Boosting; RF: Random Forest; AdaBoost, Adaptive Boosting; SVM: Support Vector Machine.

Table 5 and Fig 2 comparatively present the performance metrics of the five ML models across training and validation cohorts. Radar chart analysis revealed that in the training cohort, the RF model achieved superior predictive performance (Fig 3A), with an AUC of 0.990 (95% CI: 0.983–0.997), accuracy of 0.969 (95% CI: 0.962–0.977), sensitivity of 0.952 (95% CI: 0.930–0.974), specificity of 0.978 (95% CI: 0.976–0.980), F1-score of, 0.954 (95% CI: 0.942–0.966) and AUPRC of 0.977 (95% CI: 0.972–0.981). In the validation cohort (Fig 3B), Logistic Regression demonstrated optimal classification capability with an AUC of 0.942 (95% CI: 0.904–0.980), accuracy of 0.840 (95% CI: 0.810–0.870), sensitivity of 0.895 (95% CI: 0.843–0.947), specificity of 0.813 (95% CI: 0.755–0.870), F1-score of 0.790 (95% CI: 0.759–0.820), and AUPRC of 0.889 (95% CI: 0.867–0.911). Logistic Regression model also showed good calibration with Brier score of 0.093 (95% CI: 0.082–0.104) (Fig 2E) and clinical utility with the highest net benefit across all probability thresholds in decision curve analysis (Fig 2F). These results establish Logistic Regression as the optimal predictor for DM in GC, combining highest discriminative power (AUC), highest ability to correctly identify positive cases (recall), balanced precision-recall (AUPRC), and reliable probabilistic calibration (Brier score). The hyperparameters for 5 ML models were provided in S1 Table.

**Table 5:** A battery of metrics of five classifiers in the training and validation cohorts for fivefold nested cross-validation.

Classifier	Cohorts	AUC (95% CI)	Accuracy (95% CI)	Recall (95% CI)	Specificity (95% CI)	F1 score (95% CI)
XGBoost	Training	0.954 (0.939-0.969)	0.873 (0.857-0.890)	0.925 (0.894-0.956)	0.847 (0.810-0.885)	0.830 (0.815-0.846)
	Validation	0.935 (0.897-0.972)	0.835 (0.817-0.852)	0.884 (0.811-0.957)	0.810 (0.765-0.855)	0.780 (0.758-0.803)
Logistic	Training	0.945 (0.927-0.964)	0.863 (0.852-0.874)	0.930 (0.908-0.952)	0.829 (0.803-0.856)	0.819 (0.811-0.828)
	Validation	0.942 (0.904-0.980)	0.840 (0.810-0.870)	0.895 (0.843-0.947)	0.813 (0.755-0.870)	0.790 (0.759-0.820)
Random Forest	Training	0.990 (0.983-0.997)	0.969 (0.962-0.977)	0.952 (0.930-0.974)	0.978 (0.976-0.980)	0.954 (0.942-0.966)
	Validation	0.932 (0.893-0.972)	0.867 (0.854-0.879)	0.784 (0.727-0.841)	0.908 (0.887-0.929)	0.796 (0.771-0.820)
AdaBoost	Training	0.955 (0.940-0.970)	0.888 (0.872-0.904)	0.901 (0.861-0.941)	0.882 (0.840-0.924)	0.844 (0.831-0.857)
	Validation	0.938 (0.901-0.975)	0.861 (0.835-0.887)	0.860 (0.797-0.923)	0.862 (0.808-0.916)	0.806 (0.774-0.838)
SVM	Training	0.910 (0.886-0.935)	0.848 (0.828-0.867)	0.863 (0.821-0.905)	0.840 (0.815-0.865)	0.791 (0.764-0.817)
	Validation	0.910 (0.863-0.956)	0.836 (0.794-0.878)	0.864 (0.812-0.916)	0.822 (0.765-0.879)	0.780 (0.731-0.829)

XGBoost: eXtreme Gradient Boosting; AdaBoost, Adaptive Boosting; SVM: Support Vector Machine.

### Analysis and assessment of the Logistic regression model

**Fig 4:**
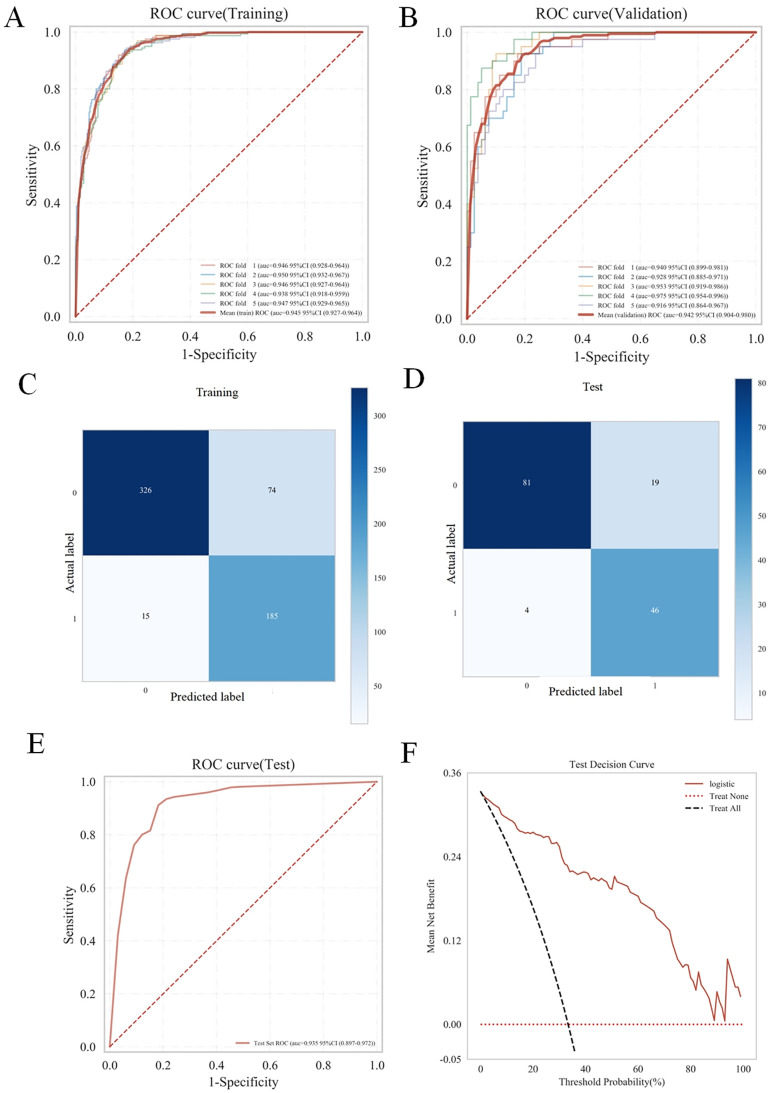
Logistic Regression performance evaluation with 5-fold nested cross-validation for gastric cancer distant metastasis prediction. The ROC curves (A, B) and confusion matrix (C, D) were conducted for Logistic Regression model within both the training and validation cohorts. (E) The calibration curve of Logistic Regression model in test cohort. (F) The decision curve analysis of Logistic Regression Model model in test cohort.

**Fig 5:**
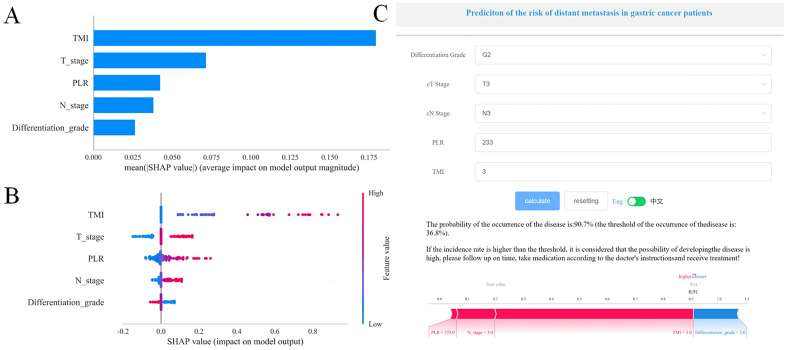
Explainability of Logistic Regression model with SHAP method and the online tool for forecasting distant metastasis in patients with gastric cancer. (A) The bar plot by SHAP. (B) The dot plot by SHAP. (C) The online tool for forecasting distant metastasis in patients with gastric cancer. Abbreviations: TMI: toumor marker index; PLR: Platelet-to-lymphocyte ratio.

As shown in Fig 4, the AUC for the validation cohort [AUC = 0.942 (95% CI: 0.904−0.980)] did not exceed that of the test cohort [AUC = 0.935 (95% CI: 0.8970.972)] by more than 10%, confirming robust model generalizability. Therefore, Logistic regression model was considered appropriate for classification tasks within this dataset. The SHAP methodology offers two distinct categories of interpretations: the bar plot visualizes the global importance of features by calculating the mean absolute SHAP value (Mean |SHAP Value|) for each feature’s impact on the model output, with features ranked by their importance. Additionally, the dot plot displays the distributional influence and directionality of features, showing how individual feature values (red indicating higher feature values and blue indicating lower values) correlate with the SHAP values across all samples, thereby highlighting both the spread and the positive/negative effects on predictions. As illustrated in Fig. 5A, the bar plot showed the influence of various features on the model presented in a descending sequence: TMI, T stage, PLR, N stage, differentiation grade. Furthermore, the dot plot (Fig. 5B) effectively depicted both the direction and extent of the impact that each feature exerts on the predictions made by the model. Notable features include advanced T and N stage, higher TMI and PLR, and poorer differentiation (Grade3, Grade4) increased the risk. From Table 6, the full model (with cT stage, cN stage, Differentiation grade, PLR, and TMI) performs best showing significantly positive IDI 0.258 to 0.309, all P < 0.001 vs. all reduced models, and TMI is the core variable boosting model performance.

**Table 6:** Comparison of integrated discrimination improvement (IDI) among models with different feature combinations.

Model	Included Features	IDI (95% CI) (Full − Reduced Model)	P-value
Reduced Model 1	cT stage + cN stage	0.309 (0.265-0.353)	**<0.001**
Reduced Model 2	cT stage + cN stage + Differentiation grade	0.304 (0.260-0.347)	**<0.001**
Reduced Model 3	cT stage + cN stage + Differentiation grade + PLR	0.258 (0.215-0.301)	**<0.001**
Full Model	cT stage + cN stage + Differentiation grade + PLR + TMI	/	/

### External test of the model and the performance differences between internal and external test

A set comprising 240 patients diagnosed with GC was assembled from the Zhejiang Provincial Hospital of Traditional Chinese Medicine for the purpose of external test. The AUC of external test was 0.879 (95% CI: 0.833–0.926).

The absolute difference in the AUC between the internal and external test sets is 0.056; The AUC of the external test showed a relative decline of approximately 6.0% compared to the internal test.

### Online prediction tool

As depicted in Fig 5C, we developed a web-based clinical decision support system (https://www.xsmartanalysis.com/model/list/predict/model/html?mid=27296&symbol=41755942uz68CdglZU92) that calculates the likelihood of metastasis by incorporating various feature variables. By applying a predefined decision threshold, the system categorizes patients into low-risk and high-risk groups. This tool combines model interpretability visualizations (SHAP force plots) with risk probability outputs to improve clinical utility and reliability.

## Discussion

This is one of the few multicenter data prediction ML models originating from a source other than the SEER database, incorporating clinical characteristics and laboratory-derived composite indices for GC with DM. It features two key innovations: 1. Transforming multiple laboratory indicators into optimized composite indices to reduce the multicollinearity issue among laboratory data; 2. Integrating sample balancing strategies and validation protocols into robust model training to mitigate the reliability and generalization limitations of imbalanced-sample models.

As of the present, various studies have reported predictive ML models that asess the risk of DM in GC; however, these studies are constrained by significant limitations. For instance, the majority of these investigations are SEER-based models, which has notable deficiencies in clinical granularity. These deficiencies include the absence of laboratory biomarkers, molecular profiles, and detailed pathological characteristics, limiting its efficacy in constructing a precise predictive model. Furthermore, the database’s population representativeness does not encompass Chinese gastric cancer sets, thereby undermining its generalizability. These limitations are corroborated through external validation in Chinese population cohorts, which revealed suboptimal discriminative performance with AUC values ranging from 0.727 to 0.760 [16–19]. Some models integrate DNA methylation profiles or miRNAs as variables; however, routine screening for genetic testing remains limited, coupled with the absence of standardized methodologies for their detection and interpretation, which severely undermines the reproducibility and generalizability of such models [29–30]. Undoubtedly, radiomics has potential value in diagnosing peritoneal metastasis of GC, However, the existing research exhibits varying levels of quality. Therefore, there is a pressing need for future research to be more standardized and of higher quality to facilitate the translation of radiomics findings into clinical practice [31–33]. Therefore, it is imperative to develop a easily data-acquirable, standardizable and clinically translatable ML model specifically tailored to Chinese populations.

Currently, The domain of medical ML faces substantial critical challenges, particularly in scenarios with small sample sizes, binary class imbalance, encompassing overfitting, constrained generalizability, and non-transparent decision-making processes, which significantly impede clinical translation [34–36]. To mitigate the impact of these issues on models’ performance, we have adopted the following relatively novel approaches: Firstly, we optimized ten single laboratory indicators into four disease-associated composite indices, which significantly reduced the model’s input dimensionality, mitigated overfitting with high-dimensional data, and helped eliminate multicollinearity among original indicators, thereby enhancing model stability and generalizability. Secondly, we employed the Borderline SMOTE-ENN method to address sample imbalance. This approach not only improves model training accuracy and more effectively captures class-specific sample distributions but also enhances the model’s adaptability to data distribution, strengthens generalization performance, and simultaneously prevents overfitting and the noise introduced by synthetic data [37]. Thirdly, we implemented a 5-fold nested cross-validation methodology to meticulously address model evaluation and hyperparameter optimization within the constraints of limited sample sizes, closely replicating real-world clinical settings. This technique operates via dual validation cycles: the outer cycle partitions the dataset into five segments, sequentially assigning one segment as an independent test cohort while the other four segments are utilized for model development. Within these four segments, the inner cycle conducts further cross-validation to adjust hyperparameters and select features, ensuring the test data remains entirely segregated from any optimization activities. This method precludes the risk of data leakage that could artificially enhance performance metrics by strictly segregating the test cohort from the model refinement stages (hyperparameter tuning, feature selection), thereby optimizing the utilization of scarce medical data. Finally, we integrated SHAP to enhance model interpretability. SHAP quantifies the contribution of each feature to individual patient predictions and visually demonstrates the directional impact (positive or negative) and magnitude of features through force-directed plots, thereby providing clinicians with an intuitive decision-making audit tool.

As a predictive model for DM, we need to comprehensively evaluate its performance with a focus on key metrics aligned with clinical needs: Recall, which reflects the model’s ability to identify patients with distant metastasis; AUC, which assesses the overall discriminative power between metastatic and non-metastatic cases; and PRAUC, which is more robust for evaluating performance in imbalanced datasets. Logistic Regression emerged as the optimal predictive model with the highest AUC, Recall, and AUPRC, while achieving the lowest Brier score. External validation across multicenter datasets yielded an AUC of 0.879, superior to existing SEER-based DM models whose external validation AUCs ranging from 0.727 to 0.76 [16–19]. This suggests that our model demonstrates strong generalization capability. The final model incorporated five critical predictors: T stage, N stage, differentiation grade, PLR and TMI. SHAP analysis revealed advanced T and N stage, high PLR and TMI, and poor differentiation markedly increased the risk. Both SHAP plots and IDI analysis confirmed that TMI is the key incremental factor in this modeling process. To address the limitations of individual TMs in diagnostic and predictive performance, numerous studies have proposed TMI as new marker, demonstrating its superior clinical utility over single-marker approaches [26–28]. In these studies, there are mainly two types of TMI calculation methods: one based on ROC and the other based on the number of positive indicators. Considering the ease of standardization for data from different populations, we adopted the latter method and successfully constructed a prediction model incorporating TMI. Our study extends the application of TMI to gastric cancer metastasis. The performance of the model incorporating TMI was significantly improved by 25.8% to 30.9% compared with models without TMI. In particular, the performance improvement was most significant when compared with the traditional T and N stages. This confirms the application value of TMI in gastric cancer.

We acknowledge certain shortcomings that require further optimization. Firstly, this investigation was retrospective in nature. Although the study implemented rigorous inclusion and exclusion criteria, completely eradicating bias from the findings proved to be a challenge. Secondly, the internal test AUC of 0.935 showed strong discriminative ability on familiar data, while the external test set AUC of 0.879 indicated slight overfitting to training data noise, though still >0.85, proving basic generalization. Future work should add diverse data to narrow this gap. Thirdly, molecular markers (E-cadherin, VEGF, and Claudin-18.2) and imaging features associated with DM in GC were not incorporated into the current research, we will integrate them in future studies. Finally, the online prediction tool for DM risk in GC developed in this study, still requires further systematic evaluation by clinicians in terms of clinical practicality, decision-making auxiliary value, and operational experience. Notwithstanding these constraints, the remarkable efficacy of our ultimate predictive model remains unaffected.

In conclusion, we developed an interpretable ML model leveraging routine EMRS data to predict the risk of DM in GC. Logistic Regression model exhibited excellent prediction ability. The online prediction tool, developed using this model, classifies patients into distinct risk categories to provide doctors with preoperative decision support.

## Supporting information

S1 Table Hyperparameters for 5 machine learning models. (DOCX)
