# Supplementary material for: Machine learning-driven risk stratification for distant metastasis in gastric cancer: A comparative study of clinical features and composite indices integrated models
Source: PLoS One. 2025 Oct 30;20(10):e0335258. doi: 10.1371/journal.pone.0335258 (PMC12574934; doi:10.1371/journal.pone.0335258)
Supplement: S1 Table — (DOCX) [file pone.0335258.s001.docx]

**S1 Table. Hyperparameters for 5 machine learning models.**

| **Models** | **Hyperparameters** |
| --- | --- |
| XGBoost | colsample_bytree: 1  learning_rate: 0.3  max_depth: 4  min_child_weight: 4  n_estimators: 5  reg_lambda: 1  subsample: 1 |
| Logistic Regression | C: 1.0  l1_ratio（ElasticNet）: None  max_iter: 50  penalty: l2  solver: lbfgs  tol: 0.0001 |
| Random Forest | criterion gini  max_depth: None  max_features: sqrt  min_impurity_decrease: 0.0  min_samples_leaf: 1  min_samples_split: 2  n_estimators: 100 |
| AdaBoost | learning_rate: 0.3  n_estimators: 50 |
| SVM | C: 0.1  gamma: scale  kernel: rbf  max_iter 50  probability: True  tol: 0.001 |
| Abbreviations: XGBoost: eXtreme Gradient Boosting; AdaBoost, Adaptive Boosting; SVM: Support Vector Machine. | |
